# Host genetics determine susceptibility to avian influenza infection and transmission dynamics

**DOI:** 10.1038/srep26787

**Published:** 2016-06-09

**Authors:** Raul Ruiz-Hernandez, William Mwangi, Marylene Peroval, Jean-Remy Sadeyen, Stephanie Ascough, Devanand Balkissoon, Karen Staines, Amy Boyd, John McCauley, Adrian Smith, Colin Butter

**Affiliations:** 1 Avian Viral Diseases program, The Pirbright Institute, Compton Laboratory, Newbury, United Kingdom; 2 Department of Zoology, University of Oxford, Oxford, United Kingdom; 3 Crick Worldwide Influenza Centre, The Francis Crick Institute, Mill Hill Laboratory, London, United Kingdom

## Abstract

Host-genetic control of influenza virus infection has been the object of little attention. In this study we determined that two inbred lines of chicken differing in their genetic background , Lines 0 and C-B12, were respectively relatively resistant and susceptible to infection with the low pathogenicity influenza virus A/Turkey/England/647/77 as defined by substantial differences in viral shedding trajectories. Resistant birds, although infected, were unable to transmit virus to contact birds, as ultimately only the presence of a sustained cloacal shedding (and not oropharyngeal shedding) was critical for transmission. Restriction of within-bird transmission of virus occurred in the resistant line, with intra-nares or cloacal infection resulting in only local shedding and failing to transmit fully through the gastro-intestinal-pulmonary tract. Resistance to infection was independent of adaptive immune responses, including the expansion of specific IFNγ secreting cells or production of influenza-specific antibody. Genetic resistance to a novel H9N2 virus was less robust, though significant differences between host genotypes were still clearly evident. The existence of host-genetic determination of the outcome of influenza infection offers tools for the further dissection of this regulation and also for understanding the mechanisms of influenza transmission within and between birds.

Influenza virus is the causative agent of influenza, a contagious respiratory viral disease of all vertebrate classes including birds and mammals. Of particular interest to the ecological landscape of human infection are viruses circulating in wild birds, domesticated poultry and pigs. The influenza viruses of avian origin (avian influenza, AI), are classified according to the basis of their ability to cause mild to severe disease in poultry as low pathogenicity avian influenza (LPAI) and high pathogenicity avian influenza (HPAI)^1^. Existing surveillance programs focus particularly on LPAI of subtypes H5 and H7, since these virus are able to mutate towards HPAI^2^,^3^ and can be responsible for direct human infections^4^. Influenza viruses of avian origin also represent an emerging threat to human health as the progenitors of the next influenza pandemic^4^,^5^,^6^.

In order to reduce the risk of the emergence of pandemic influenza from viruses of avian origin the World Health Organization have highlighted the importance of effective control measures, at the animal source together with an assessment of the animal host-specific factors related to infection^7^, as part of their research priorities. Animal models, which have been used as surrogates for evaluating human influenza infection, have contributed to the understanding of host and viral factors involved in their pathogenesis^4^ and this knowledge has been applied to the development of antiviral strategies in humans^8^. Nevertheless, compared to the contributions of viral genetic determinants on pathogenesis, which have been intensively studied, an understanding of the host contribution remains largely unexplored^9^,^10^.

Among numerous species used, the ferret model is well suited for studying both the pathogenicity and transmissibility of human and avian influenza viruses^5^,^11^, though it is limited by the lack of ferret-specific immunological reagents^4^. Mice have been widely employed to study host-genetic determinants and have helped to identify many candidates of the genetic host regions involved^9^,^12^, including the influence of Mx gene in susceptibility to influenza^10^. However, it is a poor model for virus transmission^13^. The chicken is a natural host of AIV and obviates the need to use model species.

In humans an association has been broadly described between MHC haplotypes and the outcome of infectious diseases^14^. Similarly in chickens, strong associations have been reported between the MHC haplotype (or B haplotype) and resistance and susceptibility to viral, bacterial and parasitic pathogens^15^,^16^. Although a heritable association to susceptibility to influenza in humans has been described there are no definitive reports of individual genetic variability in relation to influenza disease and its outcomes^17^. The few studies addressing this question in birds have yielded disparate results, the analysis of field infection with H5N1 HPAI suggesting a hierarchy of survival based on the B haplotype, with B21 giving complete protection from mortality^18^, whilst experimental infection of congenic lines suggested that B21 conferred only partial protection of between 60% and 30% and that non-MHC genes might also be important^19^. In the earlier study B12 appeared to be associated with high susceptibility. The discovery of the existence and mechanism of robust genetic resistance to AIV would be of substantial value in protecting birds from infection and being the source of mutated human pandemic virus. On the basis of these putative resistance and susceptibility associations we studied the infection of Line 0 birds (B21) compared with Line C-B12 (B12)^20^. Genetic resistance to infection, viral transmission, route of infection and regulation by immune responses were all subject to investigation. As the majority of incursions of avian influenza in Europe have been of H7N7 we used a virus of this serotype for the detailed analysis of genetic resistance and made comparison with two strains of H9N2 viruses that are endemic in Asia.

## Results

### Shedding trajectories and transmission of LPAI virus is dependent on host genotype

**Figure 1:**
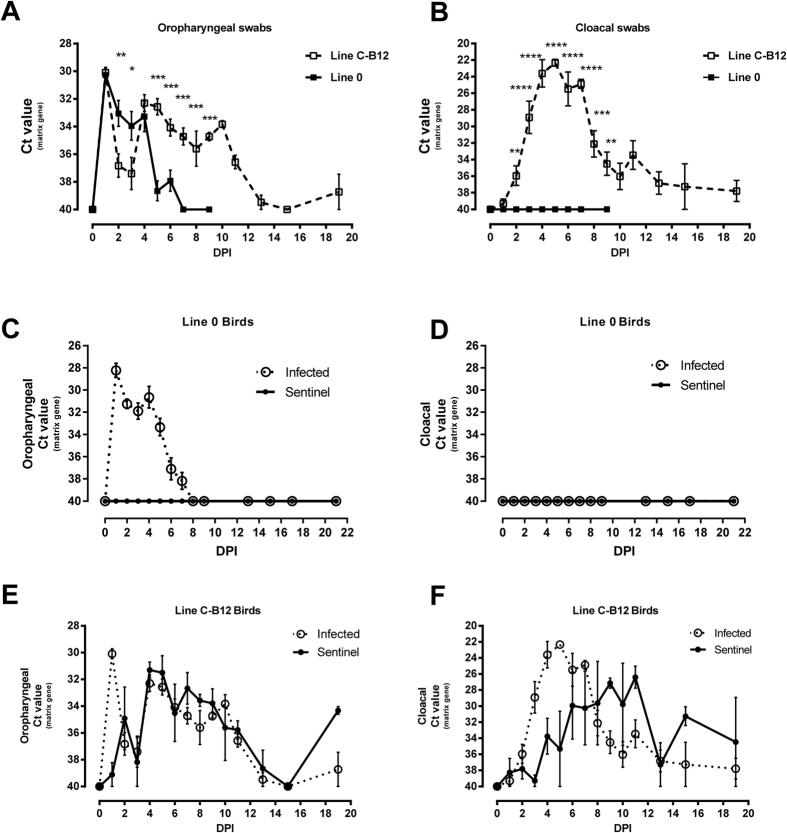
Viral Shedding trajectories from inbred lines of birds after infection with H7N7 LPAI virus. Viral shedding was measured before and during the days post-infection (DPI) by the nasel route. (**A**) data from oropharyngeal swabs of directly infected Line O birds (continuous line) and birds of line C-B12 (dashed line). (**B**) Data from cloacal swabs from the same birds. Viral shedding from sentinels housed in the same isolator (continuous line) was compared to directly infected birds (dotted line) in both Line 0 birds (**C**) oropharyngeal swabs; (**D**), cloacal swabs and C-B12 birds (**E**), oropharyngeal swabs; (**F**) cloacal swabs. Number of birds analysed: Line C-B12: directly infected Day 0–7, n = 12; days 8–13, n = 9; day 15–19, n = 5; sentinel birds n = 4; Line 0 birds (**A,B**), n = 12; (**C,D**), n = 10 sentinel and n = 10 directly infected). Results represented as mean ± S.E.M of Ct (cycle threshold) values obtained by qRT-PCR of the influenza matrix gene.

We observed clear differences in the shedding trajectories, measured by qRT-PCR, of an H7N7 LPAI virus, (A/Turkey/England/647/77) between two inbred lines of bird experimentally infected through the nares. In the case of Line 0 birds, viral shedding from the oropharynx decreased steadily from the second day post infection (DPI) and stopped one week later. Oropharyngeal shedding in Line C-B12 birds was significantly higher from day 5 post-infection (p < 0.001) and continued throughout the 3 weeks experiment (Fig. 1A). There was a complete absence of the virus in cloacal swabs from Line 0 birds, while C-B12 birds showed a clear shedding of virus from this route for the entire three week period of the experiment (Fig. 1B). These effects were independent of the viral dose, as a lower dose of infection produced similar differences in oropharyngeal and cloacal swabs (Supplementary Fig. S1).

We further confirmed the observed qRT-PCR results by analysis of infective virus by plaque assay (Supplementary Fig. S2). Virus was detected in oropharyngeal swabs in both lines, and the shedding decreased over time; the viral load and the number of positive samples in oropharyngeal swabs was significantly higher in Line C-B12 birds. The cloacal shedding of Line C-B12 birds was detected beyond two weeks after infection and not at all in Line 0.

We also detected differences in transmission of virus between lines (Fig. 1C–F): Naïve Line 0 sentinel birds co-housed with infected syngeneic birds did not shed virus (Fig. 1C,D) nor seroconvert (Supplementary Fig. S3A). However, Line C-B12 sentinel birds shed virus from the oropharynx and cloaca from 1 day after exposure to syngeneic infected birds (Fig. 1E,F and Supplementary Figs S4A,B) and all seroconverted (Supplementary Figs S3A and S4C). Directly infected birds of both lines seroconverted, with Line C-B12 birds tending to exhibit higher antibody titers (Supplementary Fig. S3). On this basis we designated Line 0 resistant and Line C-B12 susceptible to sustaining chains of infection within syngeneic populations.

**Figure 2:**
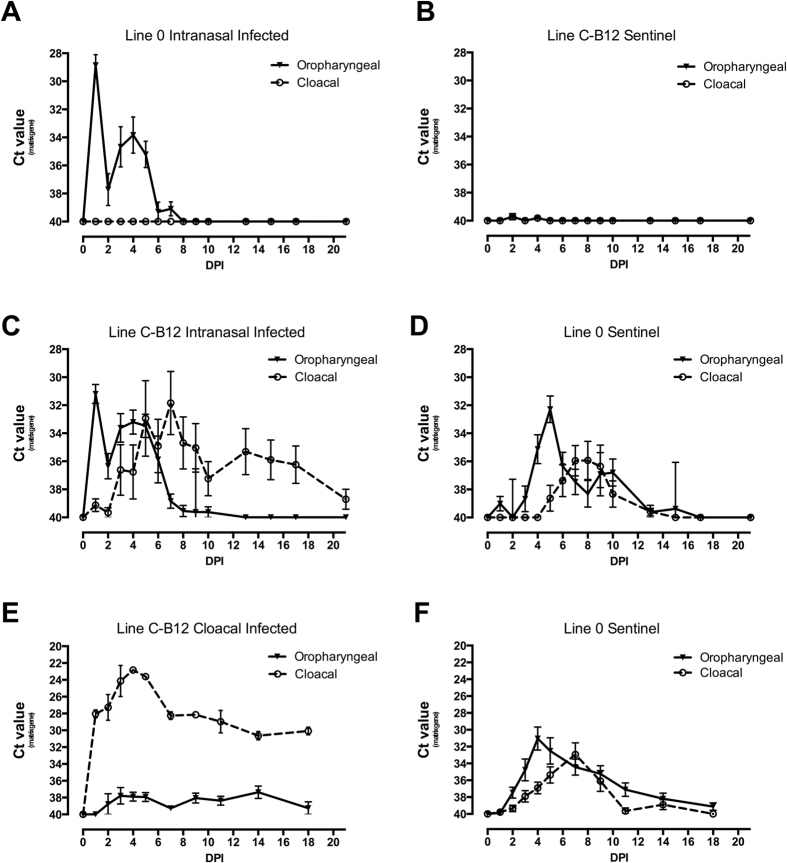
Viral shedding trajectories of sentinel birds that were in contact with birds of a different line infected with LPAI virus. Viral shedding was measured, following infection with H7N7 inoculation, in oropharyngeal (continuous line) and cloacal swabs (dashed line) of both directly-infected and contact sentinel birds. Two sets of 10 birds of each line (line C-B12 and Line 0 birds) were housed together in isolators. In the first experiment, in one isolator, only Line 0 birds were infected by the nasal route (shedding represented in (**A**)); viral shedding was analysed from contact C-B12 birds (**B**). In the other isolator, only C-B12 birds were infected (shedding represented in (**C**)) and viral shedding analysed from contact Line 0 birds (**D**). In a further experiment, 2 set of 10 birds, one of each line, were caged together and only line C-B12 birds infected by the cloacal passage ((**E**): shedding C-B12; (**F**) shedding Line 0). Results represent mean ± S.E.M of Ct (cycle threshold) value obtained by qRT-PCR of influenza matrix gene. All birds are included at each time point, irrespective of shedding status.

A subsequent experiment was performed to determine whether the virus shed from the oropharynx of the resistant Line 0 birds was capable of infecting the susceptible Line C-B12 and whether the resistant Line 0 birds were able to be infected when exposed to sustained challenge by virus shed from experimentally infected Line C-B12. Mixed populations of the two lines were housed in two isolators. In one isolator the resistant Line 0 birds were infected by the nasal route (Fig. 2A) whilst in the other the susceptible Line C-B12 birds were infected (Fig. 2C), in each case leaving the alternate line birds as uninfected sentinels (Fig. 2B,D). Infected Line 0 birds showed the characteristic oropharyngeal shedding profile, with no virus seen in cloacal swabs (Fig. 2A). None of the C-B12 sentinels produced positive swabs (Fig. 2B). In the second isolator, Line C-B12 birds shed virus by oropharyngeal and cloacal routes with the time-course previously described (Fig. 2C). All of the resistant contact Line 0 birds shed virus from the oropharynx followed, in 6/10 birds, by cloacal shedding, although this was always significantly lower than with infected C-B12 birds and over a shorter duration (Fig. 2D). 4/10 contact Line 0 birds failed to shed from the cloaca (data not shown). Seroconversion was correlated with cloacal shedding (Supplementary Fig. S3B).

We found similar results when the same susceptible Line C-B12 birds were infected via the cloacal route (Fig. 2E,F), although in this case all sentinels birds showed clear cloacal shedding.

### The magnitude of adaptive immune responses does not explain differences in susceptibility to infection

**Figure 3:**
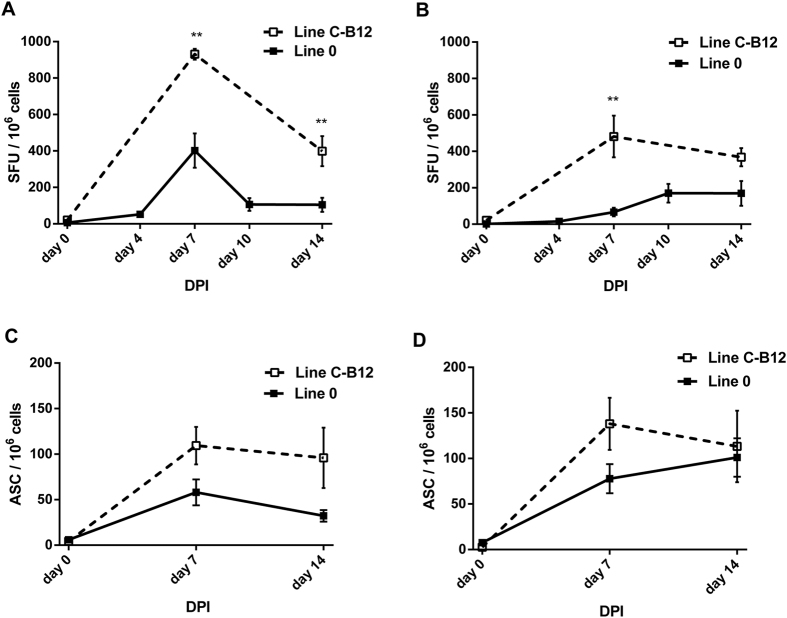
T and B cell ELISpots of splenocytes from birds infected with H7N7 LPAI virus. Following intra-nasal infection of line C-B12 (dashed line) and Line 0 (continuous line) birds, splenocytes were tested for antigen-specific induction of influenza IFNγ (**A**,**B**) and for specific influenza antibody production (**C**,**D**). A and B depict IFNγ ELISpot results represented as mean ± S.E.M of spot forming units (SFU) after cocultured with influenza infected haplotype-matched Chicken Kidney Cells, CKC (**A**) or influenza inactivated virus (**B**); all samples n = 5, except Line C-B12 (day 0, n = 3) and Line 0 (day 0 and 4, n = 4). **C,D** depict ELISpot of influenza-specific Antibody Secreting Cells producing IgM (**C**) or IgY antibody (**D**). Number of birds analysed: Line C-B12 n = 4 on day 0 and n = 5 days 7–14; Line 0 n = 6 on day 0–7 and n = 4 at day 14.

We analysed the adaptive response for a possible correlation with the different susceptibilities to infection. Splenocytes of infected birds were compared by ELISpot for detection of both antigen-specific IFNγ and antibody secreting cells (Fig. 3). Using either AIV infected syngeneic Antigen Presenting Cells (Fig. 3A) or whole inactivated virus (Fig. 3B) as recall stimulation, IFNγ responses were detected in birds of both lines after infection. At all time-points, the responses of the samples from Line C-B12 birds were higher than Line 0 birds. At day 7 postinfection, IFN responses were larger for C-B12 birds under syngeneic stimulation (930.9 ± 29.3 vs. 402.0 ± 94.2 Spot Forming Units, SFU/10^6^ splenocytes, p = 0.0079) or whole virus (481.3 ± 114.6 vs. 66.6 ± 23.6 SFU/10^6^ splenocytes, p = 0.0079). Although the values at day 14 decreased with respect to those at day 7, samples from Line C-B12 birds showed higher IFN responses at syngeneic stimulation (398.7 ± 82.8 vs. 104.5 ± 38.6 SFU/10^6^ splenocytes, p = 0.0079) or against whole virus (368.3 ± 49.9 vs. 135.8 ± 62.5 SFU/10^6^ splenocytes, p = 0.0317). We also detected influenza specific antibody IgM and IgY secreting B cells (Fig. 3C,D) in birds of both lines after infection. As with IFN, we found that responses in C-B12 birds tended to be higher than observed in Line 0 at day 7 for IgM (109.4 ± 20.6 vs. 58.0 ± 14.2 SFU/10^6^) and day 14 (95.9 ± 33.1 vs. 32.3 ± 6.4 SFU/10^6^). The number of IgY producing cells also tended to be higher at 7 DPI (131.8 ± 35.9 vs. 77.8 ± 16.0 SFU/10^6^) and 14 DPI (113.2 ± 39.2 vs. 101.0 ± 21.0 SFU/10^6^). Overall, a stronger adaptive response was consistently observed in the susceptible C-B12 birds than in resistant Line 0 birds.

### Cell autonomous responses to infection of CEF

**Figure 4:**
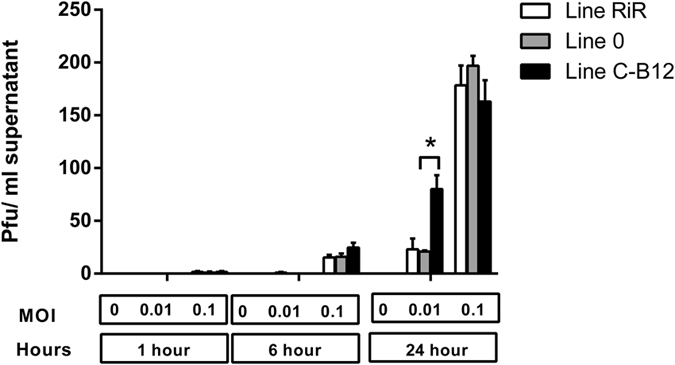
Viral production and shedding from infected CEFs from different lines of birds. Established CEF cultures of several lines (RIR = Rhode Island Red, white bars; Line C-B12, grey bars; Line 0, black bars, samples from 3 different birds per line) were infected with the H7N7 strain at different multiplicities of infection (MOI 0, 0.01 and 0.1). Supernatants of infected cultures were harvested at different times after infection and measurement of infective virus made by plaque assay. Results are given as plaque forming units (PFU)/ml of supernatants.

We compared the viral replication and infection capability of the H7N7 strain in Chicken Embryo Fibroblast (CEF) cultures from birds of Line C-B12, Line 0 and also an outbred Line of birds (Rhode Island Red, RIR). Supernatants of these infected cultures were harvested and measurement of infectious virus made by plaque assay. CEF cultures were infected at different MOI; Multiplicities of Infection (Fig. 4: MOI of 0.1, 1). Assuming that virus particles were released about 4 hours after virus absorption^21^, we took samples before one cycle of infection-replication (1 hour), after 1 cycle of infection-replication (6 hours) and several cycles of infection-replication (24 hours).

As expected, we observed that the longer the harvesting time after infection or the higher the initial multiplicity of infection, the higher the yield of infective particles. The dynamics of virus production from CEF of the three lines of bird were similar, except at 24 hours postinfection, where at the lowest dilution (MOI of 0.01) the number of infective particles produced by cells from birds of Line C-B12 (80.0 ± 13.4 pfu /ml supernatant) was higher than birds of other lines (21.0 ± 1.0 and 23.2 ± 10.0 pfu/ml supernatant in Line 0 and RIR respectively, significant at (p < 0.05)). This single time point and MOI was the only significant difference (we also tested higher MOI: 1,5 and 10, data not shown), suggesting that cell-autonomous factors were unlikely to explain the main characteristics of the resistance of Line 0 birds, the absence of cloacal shedding.

### Host factors control viral transmission within the bird

**Figure 5:**
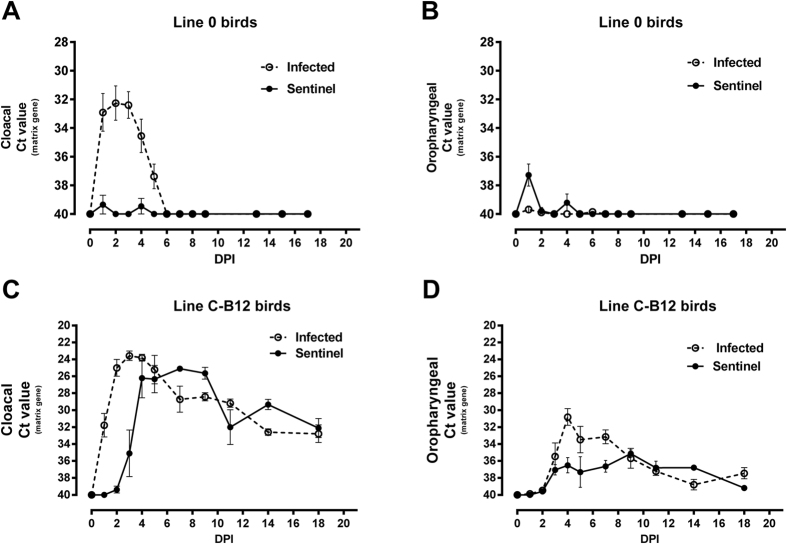
Infection with LPAI virus through the cloacal route. Birds of Line 0 (**A,B**) and line C-B12 (**C,D**), were infected with H7N7 virus by the cloacal route. Viral shedding was analysed in cloacal (**A**,**C**) or oropharyngeal swabs (**B,D**) of infected birds (continuous line, n = 10) and sentinel non-infected birds (dashed line, n = 10). Results are represented as mean (±S.E.M) of Ct (cycle threshold) value obtained by qRT-PCR of influenza matrix gene.

We hypothesised that the restricted shedding profile seen in Line 0 birds was due to a restriction in the transmission of virus from the experimentally-infected respiratory tract to the gastrointestinal tract. To test this we infected Line 0 birds by the cloacal route, in the presence of sentinel birds. Directly infected birds showed significant cloacal shedding (Fig. 5A) with a profile similar to those Line 0 birds previously infected by contact with shedding susceptible birds (Fig. 2D,F). Particularly, shedding had started to decline sharply by 4 DPI and by 6 DPI no cloacal shedding could be detected. However, viral shedding did not extend to higher respiratory tissues as no shedding was detected in oropharyngeal swabs (Fig. 5B). The serological responses of these cloacal infected birds were similar to those infected by the intra-nasal route (data not shown). Contact Line 0 birds did not show infection. By contrast, in Line C-B12 birds, transmission to sentinels was detected (Fig. 5C,D) and viral shedding extended to higher respiratory tissues (Fig. 5D).

### The ability to sustain a chain of infection of LPAI among resistant Line 0 birds correlates with cloacal shedding and is dependent on viral strain

**Figure 6:**
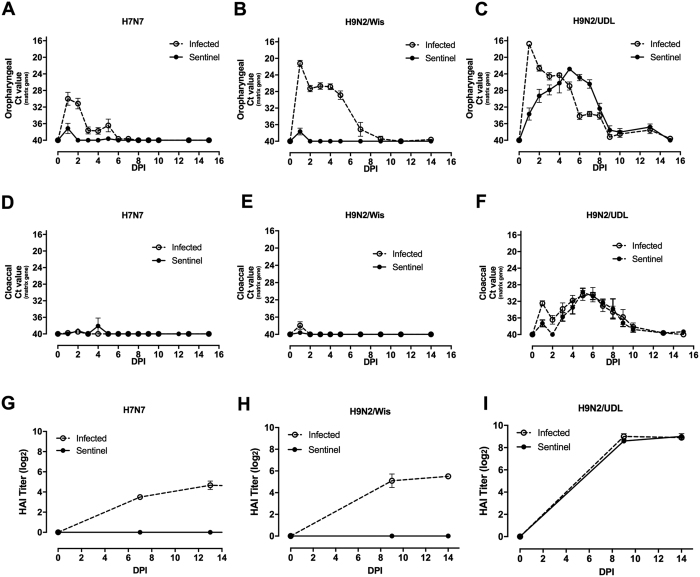
Viral shedding trajectories and antibody titres from Line 0 birds challenged with different LPAI viruses and their respective contact sentinels. Viral shedding from Line 0 birds was measured in directly infected (continuous line) or contact sentinel birds (dashed line). Oropharyngeal shedding of H7N7 (**A**), H9N2/Wis (**B**) or H9N2/UDL (**C**); cloacal viral shedding of H7N7 (**D**), H9N2/wis (**E**) or H9N2/UDL (**F**). Results are represented as the mean of Ct (cycle threshold) value obtained by qRT-PCR of the influenza matrix gene (n = 10 for each group within each virus). Influenza-specific antibody titres from serum samples from infected (continuous line) and sentinels birds (dashed line) exposed to H7N7 strain (**G**), H9N2/wis (**H**) or H9N2/UDL (**I**) were measured by haemagglutination inhibition assay and represented as mean ± S.E.M of HAI titre.

We compared the profile of viral shedding of directly infected and close-contact sentinel birds with H7N7 (A/Turkey//England/647/77) and two H9N2 LPAI strains ((A/chicken/Pakistan/UDL-02/2008 (H9N2-UDL) and A/Turkey/Wisconsin/1/66 (H9N2-Wis), Fig. 6). H7N7-infected birds showed the characteristic pattern of viral shedding, transmission of infection and serological responses previously described (Fig.6 A,D and G).

H9N2 infected birds showed significantly higher oropharyngeal shedding values (p < 0.0001) than H7N7 infected birds at any time point after infection (Fig. 6B,C), with higher values during the first day that steadily decreased, with few positives beyond day 8 post-infection. However, the two H9N2 strains were highly divergent in other aspects. Whilst H9N2/wis did not shed from the cloaca (Fig. 6E), infection with H9N2-UDL resulted in viral shedding (Fig. 6F), resolving by 10 days post-infection. Contact sentinels of the H9N2-UDL infected birds shed virus from the oropharynx and cloaca (Fig. 6C and F), with cloacal shedding almost equivalent to that of the directly infected birds. The peak of cloacal shedding was at day 5, decreasing steadily thereafter. Serological results mirrored shedding data, with all directly infected birds seroconverting but only sentinels of the H9N2/UDL infected group (Fig. 6G–I).

## Discussion

Our initial observations revealed profound differences in host-genetic control of experimental H7N7 influenza infection in chickens, including the total absence of cloacal shedding in resistant birds and a restricted time course of oropharyngeal shedding. This phenotype was maintained following infection with an historic H9N2 virus but broke down after challenge with a novel H9N2 virus. The Pakistan group of viruses to which this belongs have higher infectivity, virulence and transmission than many LPAI due to polymerases (PB2, PB1 and PA) and non-structural (NS) gene segments identical to highly pathogenic H7N3 viruses^22^,^23^. The mechanisms responsible for this increased virulence were clearly able to overcome the genetic resistance seen in Line 0 birds.

Notwithstanding reports of a degree of genetic resistance to HPAI in congenic lines of chicken^19^ and epidemiologic studies carried out in humans^17^, our finding is the most striking example so far reported of the variation of *in vivo* host-genetic control of influenza within a reservoir species that threatens the human population. Furthermore, resistant birds were completely unable to initiate or sustain a chain of infection of LPAI, with sentinel birds of either susceptible or resistant genotype showing neither infection nor seroconversion. Our consistent finding was that cloacal shedding of the directly infected birds is the main contributor to sustain a chain of transmission (Figs 1, 2 and 6). Indeed, this is the first experimental evidence of the irrelevance of oropharyngeal shedding to transmission of a particular influenza strain in intimately co-housed birds, with previous literature mostly reporting studies relying on a physical separation between infected and sentinel groups^24^. Housing resistant Line 0 birds with directly infected and shedding C-B12 birds changed their previously observed phenotype by producing a transient low level shedding of virus from the cloaca (Fig. 2D,F). Host factors were clearly still relevant as, unlike with susceptible C-B12 sentinels, not all individuals succumbed to cloacal shedding, and in those birds that were infected this stopped after 5–8 days, as compared with over two weeks for the C-B12 sentinels. Although it is possible that the change in phenotype of the resistant birds was due to persistent challenge with a high level of virus from the susceptible birds, an alternate explanation is that this was the result of an alternative route of infection. We demonstrated the possibility that infection can be achieved through the cloaca (Fig. 5), in which case the Line 0 birds reversed the phenotype seen with intra-nasel infection and shed from the cloaca and not the oropharynx, suggesting that the limited shedding trajectories seen in Line 0 birds may be due to a restriction of within-animal transmission of virus. This may be relevant to the natural situation where the virus is known to persist in water^25^, giving rise to the possibility of infection by cloacal sampling due to the Shaffner reflex^26^. Infection by an environmental route that selectively allowed cloacal shedding and onward transmission would have a profound effect on the transmission dynamics and persistence of avian influenza viruses in the wild^27^.

Though the methodology of the present study does not allow for the identification of particular genes or genomic regions that may be responsible for the observed resistance and susceptibility profiles we can infer what elements of immune regulation may be relevant. Firstly, differences in resistance and susceptibility were observed within 2–3 days postinfection, too early for an adaptive immune response and indicative of innate factors including innate immune mechanisms. However, although we cannot rule out some degree of cell-autonomous immunity there were no profound differences in viral production in *in vitro* assays (Fig. 4), suggesting that this early-acting factor, evidenced *in vivo,* was either present in cells other than the CEF used for our *in vitro* assays or was mainly exerted by a more complex level of interaction of cellular and soluble-factors. The correlation of cytokine and chemokine gene expression with viral load in influenza infected poultry has previously been described^28^. We also cannot discard the involvement of other elements triggering the immune response, such as IFN stimulated genes, TLR pathways^29^ or other genes activated in response to infection^30^. As differences in development are reflected in gene expression and the strength of host responses at transcriptional level, these factors have been linked with age-dependent susceptibility to infection in birds^28^. In the present study we also compared infection of 1 week and 3 week old Line 0 birds. However, we found the same viral dynamics and adaptive responses (data not shown) indicating that the expression of relevant host factors are independent of the host development, at least in perinatal birds.

MHC mediated adaptive immune responses have been shown to determine the resistance or susceptibility of the Compton lines of inbred chicken to a variety of pathogens^31^,^32^, including those that, like with the present study, have a very early, presumably MHC-independent, innate component of resistance^33^. Since Line 0 and C-B12 differ in their MHC haplotype, being B21 and B12 respectively, we hypothesised that adaptive responses might also be important with respect to susceptibility to infection with LPAI. However, our observations that the magnitude of adaptive response (given by IFNγ secreting cells, antibody specific clones or total influenza antibody in serum) correlates with the amount of, rather than resistance to, infection would indicate that adaptive responses may not play a significant role in the shedding/non-shedding phenotype. We cannot entirely exclude the possibility that qualitative differences in, for example, the binding capacity of MHC for specific protective viral-derived peptides may be relevant. There are also other, non-adaptive, immune mechanisms encoded within the avian MHC, including a number of chicken NK receptor genes. Polymorphic and diverse, these genes are involved in the activation or inhibition of chicken NK cells^34^,^35^,^36^. Since these cells have been considered a factor involved in avian influenza pathogenicity^37^, and that a principal activating natural killer cell receptor has been described as critical in the *in vivo* eradication of the influenza virus^38^, the importance of the B-region haplotype in relation with the NK function could be crucial. It is also possible that a hierarchy of interaction exists between the haplotype-specific NK receptors, with some haplotypes being better than others for resistance to pathogens because of increased activity of NK cells^15^,^39^. It will be important to investigate resistance and susceptibility to influenza in relation the NK activity of different haplotypes.

The present study indicates other priority areas for further investigation. The identification of the precise mechanisms and host-genetic control of resistance and non-transmissibility would offer the prospect of breeding or engineering resistant birds. Although ultimately rewarding this will be a complex undertaking as studies of genetic predisposition in mice have indicated that the host response is not only influenced by simple Mendelian inheritance of a single gene but also by complex, incompletely understood, interactions of genes and their associated variants^40^ that catalogue the global host responses to infection^9^.

Though birds resistant to influenza transmission have recently been engineered by the incorporation of interfering RNA into the avian genome^41^, there is considerable consumer resistance to this approach, while conventional selective breeding for many traits has existed since the domestication of poultry. Furthermore, as human genetic determinants for influenza are comparatively unknown^42^, a better understanding of the genes and mechanisms involved in susceptibilities identified as a result of animal models could lead to improved therapeutic options in humans^10^.

Further work will be crucial for refining understanding of host-pathogen interaction, pathogenesis and transmission of influenza in poultry, overcoming the limitation of the analysis of the human model^10^. Our work has narrowed the task of identifying and determining the host genetic factors governing influenza infection and emphasizes the importance of the host for the control of viral replication and transmission. The prospect of breeding birds with natural immunity to influenza virus would widen the scope of existing control measures and limit the risk to the human population of the emergence of pandemic viruses.

## Materials and Methods

### Ethics Statement

All studies and procedures involving animals were in strict accordance with the European and United Kingdom Home Office regulations incorporated into the Animals (Scientific Procedures) Act 1986 Amendment Regulations 2012^43^, under the authority of the Project License PPL 30/2712. Experimental protocols were approved by the Pirbright Institute Animal Welfare and Ethical Review Body.

### Experimental infection of birds

An initial stock of low pathogenic avian influenza virus strain A/Turkey/England/647/77 (H7N7) was the kind gift of Professor Ian Brown (Animal and Plant Health Agency, Weybridge, UK). A/chicken/Pakistan/UDL-02/2008 (H9N2) and A/Turkey/Wisconsin/1/66 (H9N2) strains were kindly supplied by Dr. Munir Iqbal (The Pirbright Institute). All viruses were propagated in embryonated hens’ eggs as previously described^44^.

Chickens from the Compton White Leghorn inbred lines were produced and maintained at the Pirbright Institute (Compton, UK) in specific pathogen-free (SPF) conditions and fed *ad libitum.* Groups of birds of mixed sex were housed in self-contained BioFlex^®^ B50 Rigid Body Poultry isolators (Bell isolations systems) and infected with 100 μl of influenza virus (allantoic fluid diluted in PBS) by the intranasal or cloacal routes, either with 3.4 × 10^7^ pfu/bird (high dose of infection) or 3.4 × 10^5^ pfu/bird (low dose of infection) . Birds were 3 weeks old at the time of infection unless otherwise stated. Additional uninfected control birds were kept in a separate room.

### Experimental infection of Chicken Embryo Fibroblasts

Chicken cell lines were produced and maintained at the Pirbright Institute (Compton, UK). Chicken Embryo Fibroblasts (CEFs) of 3 different birds each of Lines C-B12 and 0 were harvested from 10-day old embryos, washed and grown in T25 flasks (Nunc) in a culture media containing Medium 199 supplemented with TPB Glutamine (Sigma), 100 U/ml Penicillin, 100 μg/ml streptomycin and 10% heat inactivated fetal bovine serum (FBS), equilibrated to neutral pH with sodium bicarbonate and maintained in 5% CO_2_ at 41 °C. Confluent monolayers of cells were washed with warm PBS and infected with a solution of culture media (without FBS) 0.5 μg/ml TPCK trypsin (Sigma) containing H7N7 strain at different Multiplicity of Infection (MOI) of 0.1 and 0.01. After 1 hour incubation at 41 °C, cells were washed again and culture media solution was added to the monolayers. After incubation at 1, 6 and 24 hours at 41 °C, supernatant of infected cells were harvested, centrifuged at 300 × g for 10 min to remove dead cells and frozen at −80 °C.

### Determination of viral titers

Determination of viral load was performed by detection of viral matrix mRNA transcript by qRT-PCR of swabs as previously described^44^,^45^, or by plaque assay plaque assay on Madin-Darby canine kidney (MDCK) cells by standard methods^46^. MDCKs were supplied by the microbiological services unit at the Pirbright Institute (Compton, UK).

### Determination of adaptive response

Determination of haemagglutination titres and IFNγ ELISpot assays of splenocytes cocultured with influenza infected haplotype-matched CKC or influenza inactivated virus was carried out as previously described^45^. For antigen-specific B Cell ELISpots, MultiScreen™-HA ELISPOT plates (Millipore, UK) were coated for 2 h at 37 °C with 2 μg /mL of anti-H7 and anti-N7 antibodies (2B scientific) in carbonate buffer (Sigma). Plates were washed with PBS and subsequently incubated for another 2 h at 37 °C with 100 μL per well of blocking buffer (PBS containing 4% dried skimmed milk). After the PBS wash, a suspension in PBS of 2 × 10^6^ PFU per well of the viral strain employed for challenge was added and incubated for 2 h at 37 °C After a PBS wash 5 × 10^5^ splenocytes were added per well and cultured overnight at 37 °C with 5% CO_2_ atmosphere. Cells were washed off the plates using PBS containing 0.05% Tween20, and 100 μL of biotinylated goat anti-chicken IgM (antibodies-online.com) or biotinylated goat anti-chicken IgY (Vector Laboratories) diluted in PBS at 2 μg/ml added per well. After a 3 h incubation at room temperature plates were washed with PBS including 0.05% Tween20 and 100 μL per well of avidin/biotinylated enzyme complex (Vectastain Elite ABC kit, Vector Laboratories) added. Following 30 min incubation at room temperature and a PBS wash, 100 μL per well of 3-amino-9-ethylcarbazole (AEC) substrate (Merck Chemicals, UK) were added at room temperature. After spots had appeared, plates were rinsed with tap water and allowed to dry overnight at room temperature before counting the red-coloured immunospots using an AID ELISpot reader. Results were expressed as ASC (antibody secreting cells) number per 10^6^ splenocytes.

### Statistical analysis

A Mann-Whitney (two-tailed) test was performed using GraphPad Prism (GraphPad Software, San Diego California USA). Significance of the data is represented as follows: *p < 0.05, **p < 0.01, ***p < 0.001, ****p < 0.0001.

## Additional Information

**How to cite this article**: Ruiz-Hernandez, R. *et al*. Host genetics determine susceptibility to avian influenza infection and transmission dynamics. *Sci. Rep.* **6**, 26787; doi: 10.1038/srep26787 (2016).

## Supplementary Material

Supplementary Information
