# Supplementary material for: Host genetics determine susceptibility to avian influenza infection and transmission dynamics
Source: Sci Rep. 2016 Jun 9;6:26787. doi: 10.1038/srep26787 (PMC4899695; doi:10.1038/srep26787)
Supplement: Supplementary Information [file srep26787-s1.pdf]

Host genetics determine susceptibility to influenza infection and transmission dynamics

Raul Ruiz-Hernandez[1], William Mwangi[1], Marylene Peroval[1], Jean-Remy Sadeyen[1], Stephanie Ascough[1] , Devanand Balkissoon[1], Karen Staines[1,4], Amy Boyd[2], John McCauley[3], Adrian Smith[2], Colin Butter[1,4]*

[1] Avian Viral Diseases program, Compton Laboratory, Newbury, The Pirbright Institute, United Kingdom

[2] Department of Zoology, University of Oxford, Oxford, United Kingdom

[3] Crick Worldwide Influenza Centre, The Francis Crick Institute, Mill Hill Laboratory, London, United Kingdom

[4] Present address: - School of Life Sciences, University of Lincoln, Joseph Banks Laboratories, Green Lane, Lincoln, United Kingdom

* Corresponding author: Dr Colin Butter

School of Life Sciences, University of Lincoln, Joseph Banks Laboratories, Green Lane, Lincoln, LN6 7DL, United Kingdom,

Tel: +44 7973 381086

E-mail:colin.butter@avianimmunology.org

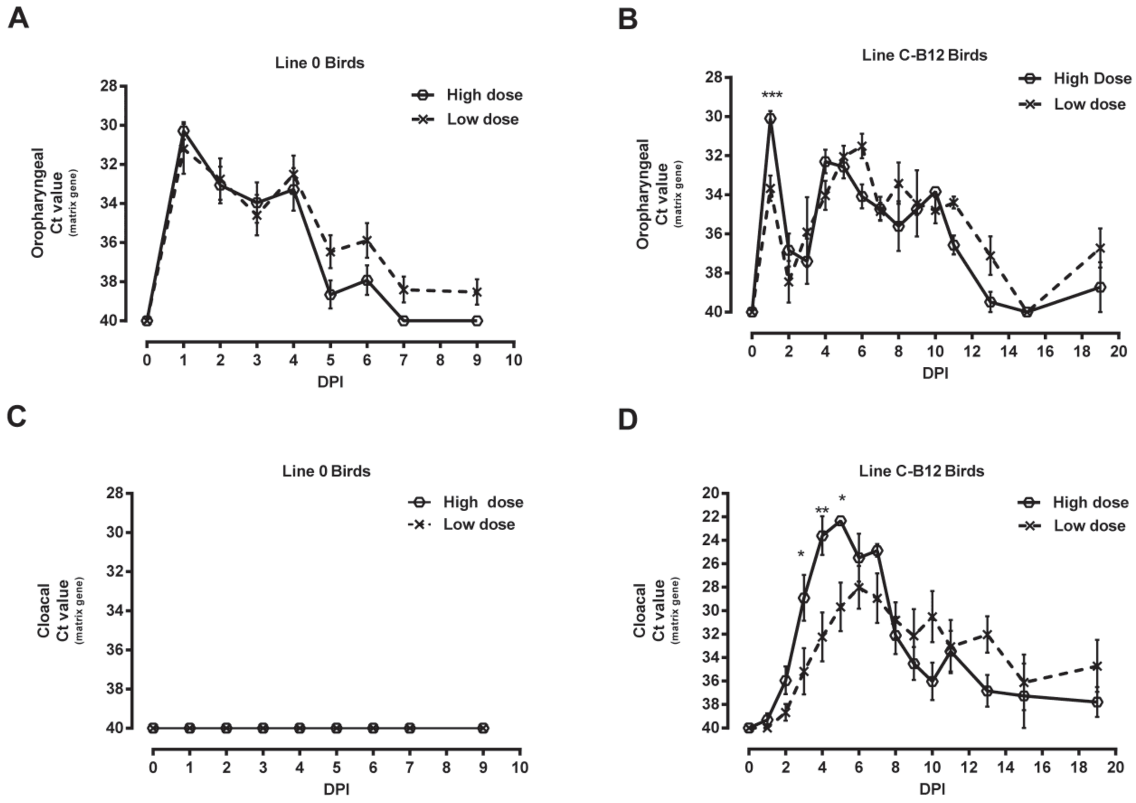

Figure S1 Viral shedding trajectories are independent of the dose of infection with a LPAI virus. Following inoculation with a high dose (3.4 x10^7 pfu/ bird) or low dose (3.4 x10^5 pfu/ bird) of the LPAI H7N7 strain, determination of viral shedding was performed in swabs taken from birds before and during the days post-infection (DPI). Figure shows viral shedding in samples from birds infected with a high dose (continuous line) or low dose (dashed line). (A) Oropharyngeal shedding Line 0 birds; (B) Oropharyngeal shedding line C-B12 birds; (C) Cloacal shedding in Line 0 birds; (D) Cloacal shedding in line C-B12 birds; Number of birds analysed in each time point: Line C-B12: Day 0-7, n=12; days 8-13, n=9; day 15-19, n= 5. Line 0, n=12. Results represented as mean (±S.E.M) of Ct (cycle threshold) values obtained by RT-PCR Real Time of influenza matrix gene.

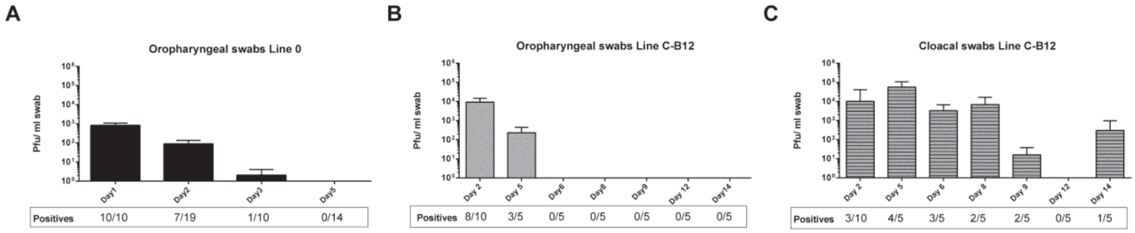

Figure S2. Detection of production of infective viral particles from swabs of infected birds. Following inoculation with a high dose of infection of the H7N7 strain, determination of viral shedding was performed by plaque assay of swab supernatants at the different days post infection (x Axis) of the oropharyngeal swabs of Line 0 birds (A); oropharyngeal (B) and cloacal swabs (C) from line C-B12. Results given as plaque forming units (PFU) per ml of swab. For all graphs, of the given numbers positioned under each analysed day, right number indicated the number of samples analysed and left number indicates number of positives for presence of viral infecting particles.

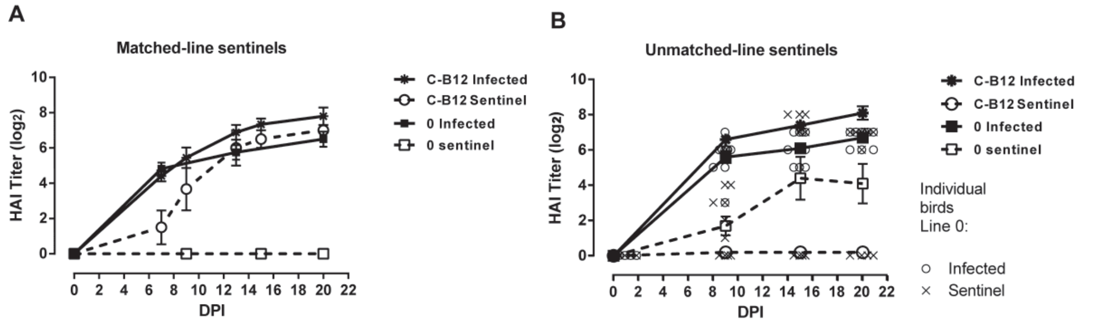

Figure S3. Antibody titres from inbred lines birds after infection with H7N7 strain. Following infection with high dose of infection of the H7N7 strain of line C-B12 and Line 0 birds, serum samples from infected birds (continuous line) and either sentinels line matched (A) or unmatched (B) sentinels birds (dashed line) were taken and antibody titres against influenza were semi quantified by haemagglutination inhibition assay. (A) Line 0 birds, both sentinel and infected birds n=10; Line C-B12 birds : infected group day 0-7, n=16; day 9-15, n=12; day 13-20, n=6; sentinel group, n=4. (B) The figure represents serum samples from Line 0 birds (both sentinel and inoculated birds n=10) and

line C-B12 birds (n=10 for each group of sentinels and infected birds). In the case of Line 0 birds, data from directly infected Line 0 birds (circles) and sentinel in contact with infected C-B12 birds (cross) shows that only some unmatched line sentinels birds developed serological responses. Results are represented as mean ±S.E.M of HaemAgglutination Inhibition units (HAI titer).

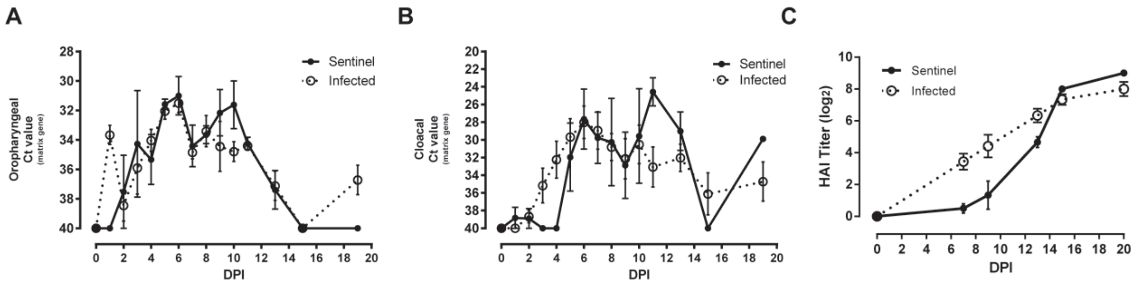

Figure S4. Viral shedding trajectories of sentinels birds and infected C-B12 **birds with a low dose of infection.**

Following infection with a lower dose of the H7N7 influenza strain, viral shedding from sentinels birds (continuous line) housed in the same isolator that held the influenza-infected birds was measured by detection of influenza matrix gene by RT-PCR in swabs taken before and during the days post-infection (DPI). Viral shedding from sentinels (continuous line) was compared to those directly infected birds (dotted line) in both Line C-B12 birds (A: oropharyngeal swabs; B: cloacal swabs). Number of birds analysed: day 0-7, n=16; day 9-15, n=12; day 13-20, n=6 of directly infected group; sentinel birds n=4). Results represent as mean ±S.E.M of Ct (cycle threshold) or HaemAgglutination Inhibition units (HAI titer, Fig. C).
